# Prognostic value of the immunohistochemical score based on four markers in head and neck squamous cell carcinoma

**DOI:** 10.3389/fimmu.2023.1076890

**Published:** 2023-02-22

**Authors:** Qing-Qing Xu, Qing-Jie Li, Zhen Xu, Li-Long Lan, Zan Hou, Juan Liu, LiXia Lu, Yuan-Yuan Chen, Run-Zhe Chen, Xin Wen

**Affiliations:** ^1^ Department of Radiation Oncology, Nanfang Hospital, Southern Medical University, Guangzhou, China; ^2^ Department of Radiation Oncology, Sun Yat-sen University Cancer Center, State Key Laboratory of Oncology in South China, Collaborative Innovation Center for Cancer Medicine, Guangdong Key Laboratory of Nasopharyngeal Carcinoma Diagnosis and Therapy, Guangzhou, China; ^3^ Department of Radiation Oncology, The First Affiliated Hospital, Sun Yat-Sen University, Guangzhou, China

**Keywords:** head and neck squamous cell carcinoma, immunohistochemistry, nomogram, prognosis, TNM system

## Abstract

**Purpose:**

Head and neck squamous cell carcinoma (HNSCC) ranks sixth among all cancers globally regarding morbidity, and it has a poor prognosis, high mortality, and highly aggressive properties. In this study, we established a model for predicting prognosis based on immunohistochemical (IHC) scores.

**Methods:**

Data on 402 HNSCC cases were collected, the glmnet Cox proportional hazards model was used, risk factors were analyzed for predicting the prognosis of survival, and the IHC score was established. We used the IHC score to predict disease-free survival (DFS) using training and independent validation cohorts, including 264 cases in total. Additionally, the accuracy of the IHC score and the TNM system (8^th^ edition) was compared. A DFS prediction nomogram was established by combining the prognostic factors.

**Results:**

The IHC scores included CK, Ki-67, p16, and p40 staining intensity. The concordance index and the Kaplan-Meier survival analysis showed that the IHC scores had high predictive power for HNSCC. Our results showed that the IHC score is an independent factor that can predict prognosis in a multivariate Cox regression analysis. When predicting DFS, the IHC score had a significantly higher value for the area under the ROC curve (AUC) than that of the TNM system. A nomogram was established and included the IHC score, age, tumor location, and the TNM stage. The calibration curves exhibited high consistency between the prognosis predicted by our nomogram and the actual prognosis.

**Conclusions:**

The IHC score was more accurate than the eighth edition of the TNM system in predicting HNSCC prognosis. Therefore, combining the two methods can facilitate individualized patient consultation and care.

## Introduction

Head and neck squamous cell carcinoma (HNSCC) includes nasopharyngeal cancer, oral cavity and oropharyngeal cancer, as well as laryngeal and hypopharyngeal cancer (1, 2). HNSCC is mainly treated by surgical resection, chemotherapy, and radiotherapy (3). Despite the development of comprehensive therapeutic strategies, a high recurrence rate and metastasis hinder the effective treatment of HNSCC (4). Therefore, early prognostic prediction and subsequent individualized treatment strategies are ideal for treating HNSCC patients. HNSCC is treated, and its prognosis is predicted mainly using the TNM classification system (8^th^ edition) published by the American Joint Committee on Cancer (AJCC) (5). However, individual heterogeneity causes inaccurate prognosis prediction (6). Therefore, more accurate predictors are required.

As an inexpensive and convenient pathological technique, immunohistochemistry (IHC) is commonly used to analyze the occurrence, development, and invasiveness of HNSCC. Many studies have shown that IHC is an efficient approach for predicting survival in cancer patients. For example, cytokeratin (CK) is a specific marker of oral squamous epithelial cells and has a poor prognosis with oral squamous cell carcinoma (7, 8). Ki-67 is an immunohistochemical marker of cell proliferation and a predictor of local recurrence in papillary thyroid cancer (9). The protein p16Ink4a (p16) is a tumor suppressor protein that is underexpressed in different types of cancers, like bladder cancer, breast cancer, sarcoma, glioblastoma, lung cancer, colonic cancer, and hematologic neoplasms (10–12). The p16 protein is associated with a favorable prognosis (13). The p40 protein is an important prognostic factor for meningioma recurrence (14). The p53 protein is a ubiquitous protein in humans and a well-known tumor suppressor gene. Mutation or overexpression of the p53 gene is quite common in HNSCC (15, 16), but the prognostic significance of p53 is debatable (17, 18). The immunohistochemical marker p63 is a myoepithelial marker (19). The overexpression of p63 is associated with poor prognosis in oral cancer (20). Although IHC markers can predict tumor prognosis, a model based on multiple independent IHC prognostic factors is still lacking in HNSCC.

Therefore, in this study, we used the lmnet Cox proportional hazards model for constructing an IHC score to predict DFS in HNSCC patients. The predictive capacity of IHC score was validated using the concordance index (C-index) and by performing the Kaplan-Meier survival analysis. Additionally, a nomogram for the combined IHC score and other clinicopathological risk factors was established. This nomogram might be used for guiding management decisions in patients with HNSCC.

## Materials and methods

### Patients

**Figure 1:**
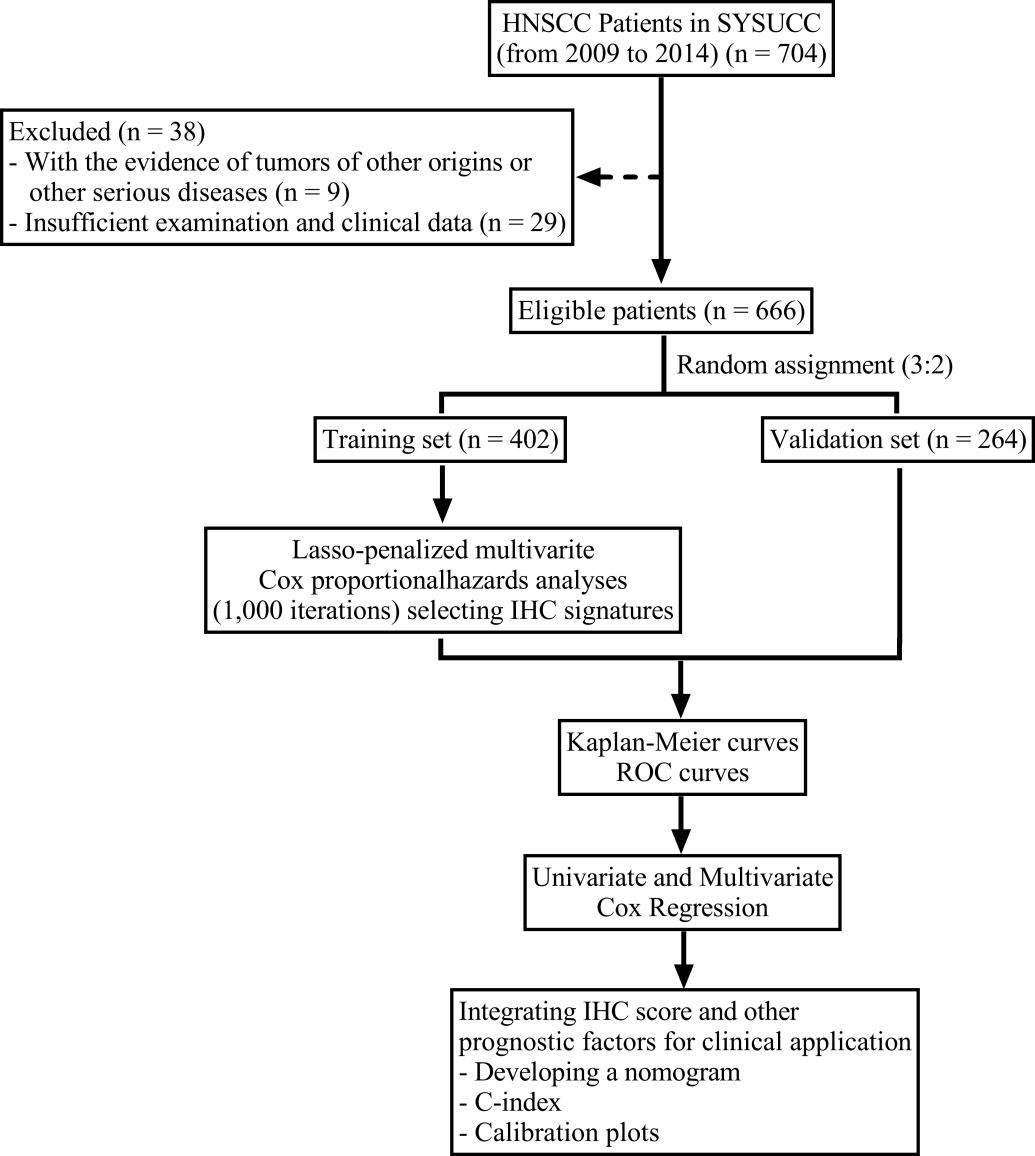
A flowchart of the study design.

From January 2009 to June 2014, the data on 666 patients with *de novo* HNSCC were collected from Sun Yat-Sen University Cancer Center. The inclusion criteria were: 1. Histopathologically confirmed non-metastatic HNSCC patients. 2. No evidence of tumors of other origins or other serious diseases. The exclusion criteria were: 1. Without tissue sections available for further immunohistochemistry staining. 2. Insufficient follow up data. The cases were randomized as training (n = 402) and validation (n = 264) cohorts at a ratio of 3:2. The flowchart for patient eligibility is presented in **Figure 1**. Information on age, sex, drinking status, smoking status, and tumor location of all patients was obtained from the clinical data. We re-recorded tumor staging for each patient using the TNM classification system (8^th^ edition). The Institutional Review Board of Sun Yat-Sen University Cancer Center approved this study. No informed consent was required since we examined anonymous data.

### Follow up

In this study, the disease-free survival (DFS) was considered to be the primary endpoint, which indicated the duration between the initial diagnosis date and the relapse or final follow-up date. All patients visited the clinic for follow-up at three-month intervals for the first 1-2 years, six-month intervals for the next 3-5 years, and one-year intervals thereafter till the last follow-up or death.

### IHC staining and evaluation

The IHC assays of all patients were conducted at the Department of Pathology in our center. All specimens were tissue sections that were fixed in formalin and embedded in paraffin. The primary antibodies used were pan-cytokeratin (CK) (Santa Cruz, 1:500), Ki-67 (Dako, 1:100), p16 (Ventana, 1:500), p53 (Ventana, 1:500), p40 (Biocare, 1:100), and p63 (Dako, 1:100). The secondary antibody reagent used was a True Envision HRP Rabbit/Mouse Detection System (Dako Real Envision Systems). The sections were prepared using the 3,3’-diaminobenzidine stain, hematoxylin was used for control staining, and the rabbit immunoglobulin G (IgG; r&amp, d, Germany) or mouse IgG (Dako Cytomation, Glostrup, Denmark) was used as a negative control. The results of the IHC assay for all markers were semiquantitatively evaluated by two trained pathologists independently in a blinded manner. The staining intensity was categorized as positive (moderate/strong staining) or negative (weak/no staining), which depended on the reactivity of the IHC marker.

### Establishment and validation of the IHC score

The data from 402 HNSCC cases were used as the training cohort to establish the IHC score. We used the “glmnet” package in the R software to determine the optimal model based on the Cox proportional hazards model under lasso penalty with 1,000 iterations (21). Then, the IHC score was established using the as-constructed optimal gene model. Later, the prediction ability of the signatures in the training and independent validation cohorts (264 cases) was verified by the concordance index (C-index) proposed by Harrell et al. (22).

The threshold for the low and high IHC score groups was determined based on the ROC survival analysis. Moreover, for graphically presenting the DFS of the low and high IHC score groups, we plotted the Kaplan-Meier (K-M) curves. The Cox regression analysis was performed to determine the prognostic significance of the IHC score.

### Establishing and assessing an IHC score-based nomogram

Using the rms package in R, the IHC score-based nomograms were established based on all the identified factors that independently predicted prognosis. The C-index (with a 95% confidence interval) was used for determining the nomogram prediction performance of the training and validation cohorts. One-year and three-year DFS calibration plots were generated to compare the predicted DFS with the actual DFS. The IBM SPSS25.0 and the R software (version 4.2.0) were used for performing statistical analyses.

## Results

### Patient characteristics

**Figure 2:**
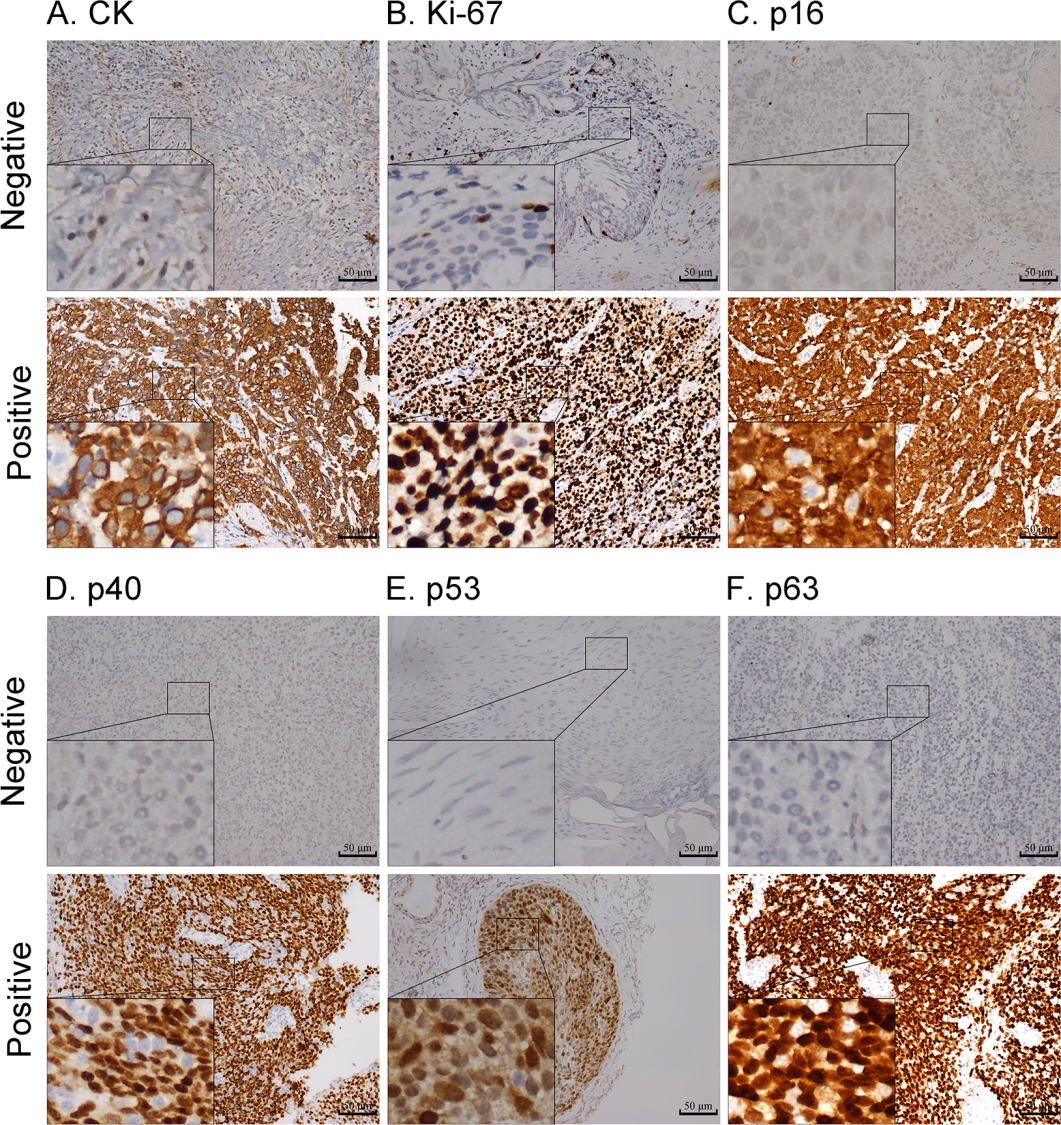
Representative images of negative and positive expression of CK **(A)**, Ki-67 **(B)**, p16 **(C)**, p40 **(D)**, p53 **(E)**, and p63 **(F)** markers; magnification: 200x. Highly magnified images (800x) are shown in the inset (scale bar = 50 µm).

We placed 402 and 264 cases into the training and validation cohorts, respectively. For the two cohorts, the ages were 20–89 (median = 59) and 18–90 (median = 58) years, respectively. The patients were followed up for 3–198 (median = 30) months, and 281 individuals experienced relapse or death. The three-year DFS rates of the two cohorts were 62.1% and 61.7%, respectively. The comparison of the baseline features between the two cohorts is presented in **Table 1**; the baseline features were similar in the training and validation cohorts. The representative immunohistochemical sections of the six IHC signatures (CK, Ki-67, p16, p40, p53, and p63) are shown in **Figure 2**.

**Table 1:** The baseline features of the training and validation cohorts.

Characteristic	Number of patients (%)		*P*-value
	Training cohort (n = 402)	Validation cohort (n = 264)	
Gender			
Male	325 (80.85%)	220 (83.33%)	0.415
Female	77 (19.15%)	44 (16.67%)	
Age (years old)			
< 60	215 (53.48%)	143 (54.17%)	0.862
≥ 60	187 (46.52%)	121 (45.83%)	
Smoking status			
No	207 (51.49%)	135 (51.14%)	0.928
Yes	195 (48.51%)	129 (48.86%)	
Drinking status			
No	290 (72.14%)	183 (69.32%)	0.432
Yes	112 (27.86%)	81 (30.68%)	
Tumor location			
Mouth	163 (40.55%)	112 (42.42%)	0.94
Pharynx	71 (17.66%)	47 (17.8%)	
Larynx	130 (32.34%)	83 (31.44%)	
Hypopharynx	38 (9.45%)	22 (8.33%)	
T stage			
T1	93 (23.13%)	59 (22.35%)	0.908
T2	147 (36.57%)	97 (36.74%)	
T3	71 (17.66%)	52 (19.7%)	
T4	91 (22.64%)	56 (21.21%)	
N stage			
N0	200 (49.75%)	121 (45.83%)	0.709
N1	63 (15.67%)	44 (16.67%)	
N2	126 (31.35%)	92 (34.85%)	
N3	13 (3.23%)	7 (2.65%)	
M stage			
M0	381 (94.78%)	246 (93.18%)	0.391
M1	21 (5.22%)	18 (6.82%)	

All continuous variables were changed to categorical variables.

Pearson χ^2^ test was used to compute the P-value.

### Construction of the IHC score

**Figure 3:**
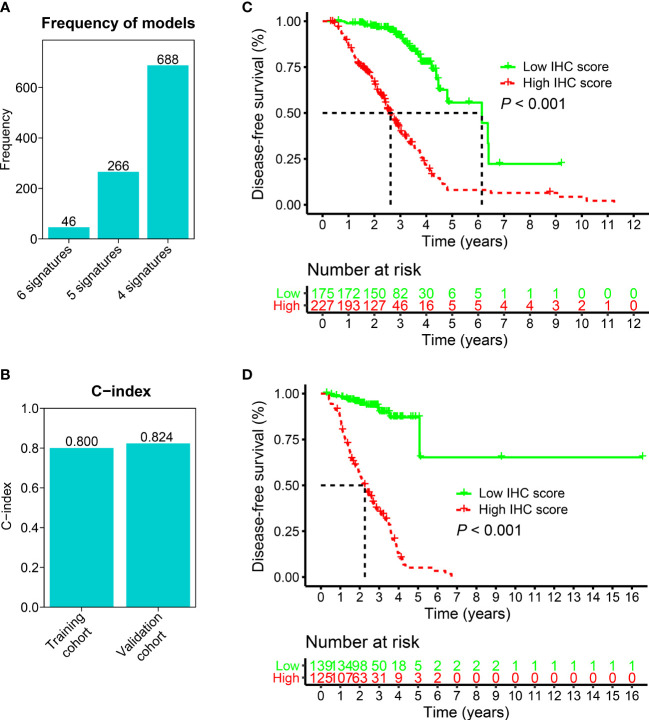
The construction and validation of the IHC score. **(A)** Generation of three models after 1,000 iterations. The model consisting of four IHC markers and with the highest frequency (688) was used to build the IHC score. 6 signatures: CK, Ki-67, p16, p40, p53, and p63; 5 signatures: CK, Ki-67, p16, p40, p53; 4 signatures: CK, Ki-67, p16, and p40. **(B)** For training and validation cohorts, the C-index values were 0.800 and 0.824, respectively. **(C, D)** K-M analyses were conducted on the IHC scores for the training and validation cohorts. In the training **(C)** and validation **(D)** cohorts, patients who had higher IHC scores showed poorer DFS, which suggested that the IHC score was a strong predictor of survival outcomes in the HNSCC patients (p< 0.001).


The Cox proportional hazards regression model was applied to six IHC markers (CK, Ki-67, p16, p40, p53, and p63) under 10-fold cross-validation to generate the optimal model. A total of 1,000 iterations were conducted, including three models for further screening. As shown in **Figure 3A**, the model with four IHC markers (CK, Ki-67, p16, and p40) had the highest (n = 688) frequency. This model was considered to be the optimal model for generating the IHC score regarding HNSCC. Thus, four IHC markers in this model were adopted to construct the IHC score. The coefficients of CK, Ki-67, p16, and p40 obtained from the LASSO Cox regression were used to construct the formula of IHC score:

IHC score = 0.403*CK + 0.448*Ki−67 – 1.715*p16 + 0.632*p40

In the above formula, the IHC staining was scored as negative (score 0), and positive (score 1).

### Validation of the IHC score

**Figure 4:**
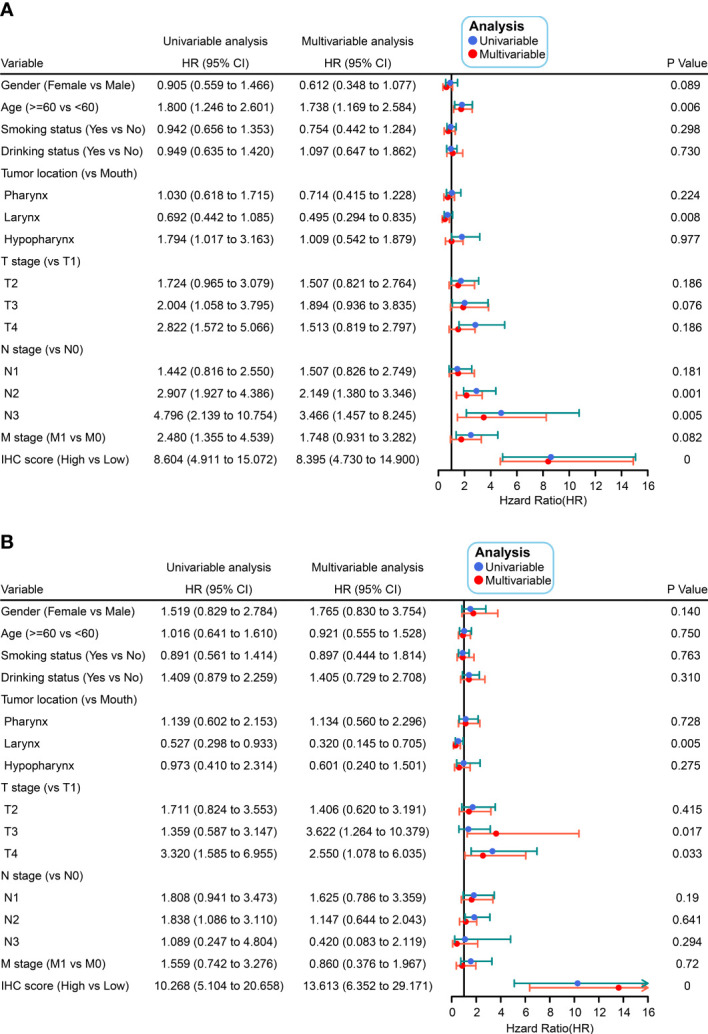
Univariate and multivariate Cox analyses were conducted on IHC scores as a function of clinicopathological characteristics in the training **(A)** and validation **(B)** cohorts.

To validate the IHC score, we predicted the DFS by calculating the C-index. The results showed that the C-index values associated with the IHC score for training and validation cohorts were 0.800 and 0.824, respectively (p< 0.05, **Figure 3B**). This indicated that the IHC score had high predictive accuracy for survival. Then, based on the ROC threshold, all cases were classified into low and high IHC score groups. Patients with high IHC scores showed a lower DFS than those with low IHC scores in both the training and validation cohorts (**Figures 3C, D**). The results of the univariate analysis of IHC scores also revealed that the IHC scores were significantly related to the DFS for the HNSCC cases in both training and validation cohorts (p< 0.05, **Figure 4**). The results of the multivariate Cox regression analysis showed that the IHC score served as an independent factor for predicting the patient prognosis after clinicopathological characteristics like age, sex, smoking history, drinking history, tumor location, and the TNM stage were adjusted in the training cohort [Hazard ratio (HR) = 8.395, 95% confidence intervals (CI): 4.730–14.900, P< 0.001] and the validation cohort (HR = 13.613, 95% CI: 6.352–29.171, P< 0.001) (**Figure 4**).

### Comparison of the IHC score with the TNM system

**Figure 5:**
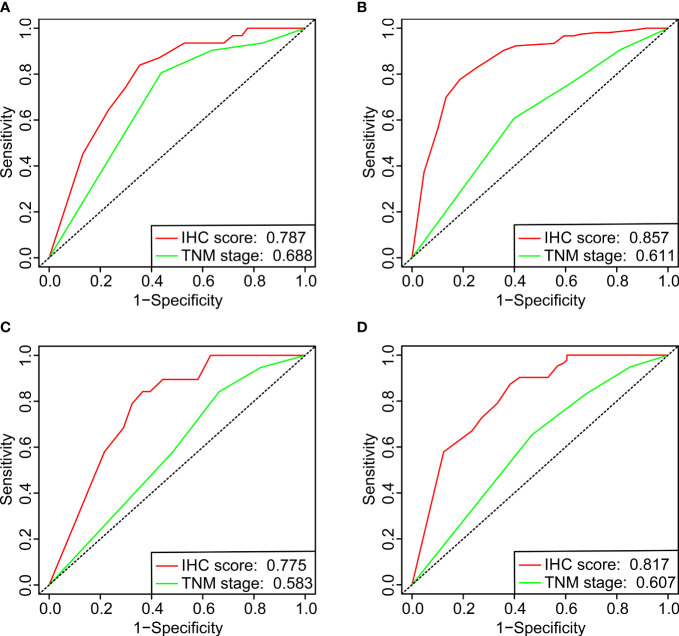
The ROC curves of one-year **(A, C)** and three-year **(B, D)** DFS for comparing the IHC scores with the UICC/AJCC TNM classification system (8^th^ edition) in the training **(A, B)** and validation **(C, D)** cohorts.

The prediction accuracy of the IHC score and the TNM system (8^th^ edition) was compared for the DFS between the two cohorts. The values of the area under the ROC curve (AUC) for the IHC score related to the prediction of the one-year and three-year DFS were 0.787/0.857 and 0.775/0.817 for the training and validation cohorts, respectively, which were considerably higher than those of the TNM stage (**Figure 5**). The results showed that the prediction accuracy and the discriminative power of the IHC score for DFS were significantly higher than that of the TNM system.

### Establishment and verification of a nomogram

**Figure 6:**
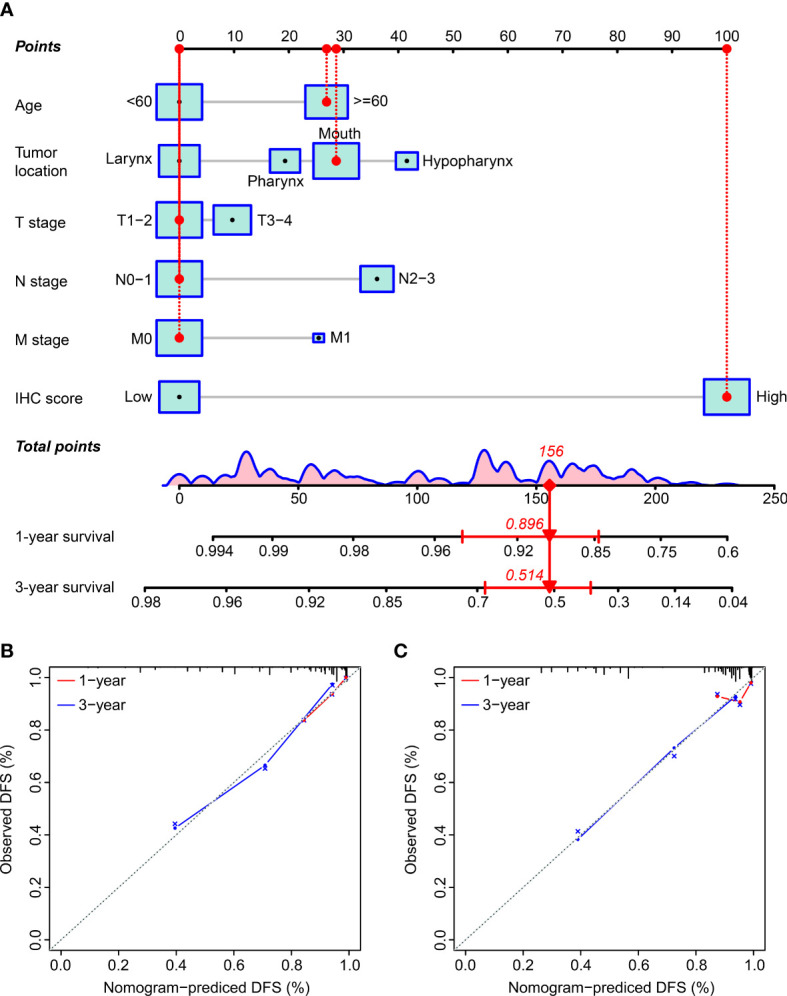
The establishment and verification of the nomogram. **(A)** The nomogram incorporated the IHC score and clinicopathological factors. **(B, C)** The predictive power of the nomogram was assessed for the training **(B)** and validation cohorts **(C)** based on the one-year and three-year DFS using the calibration curves. The dashed line below the line at an angle of 45° indicates the equivalent nomogram-predicted probability and the measured counterpart.

We established a nomogram by incorporating key prognostic factors, including the IHC score, age, tumor location, T stage, N stage, and M stage, to provide a quantitative tool for clinically predicting the probability of DFS in HNSCC patients (**Figure 6A**). The C-index values for predicting the DFS were estimated to be 0.803 and 0.800 (95% CI: 0.768–0.838 and 0.747–0.853), respectively, for the training and validation cohorts. The C-index in all the cohorts was greater than 0.7, indicating that the model was satisfactory. Calibration plots for the one-year and three-year DFS probabilities also showed significant correspondence between predictions and observations for all cohorts (**Figures 6B, C**).

## Discussion

HNSCC shows high aggressiveness and poor prognosis. Significant progress has been made in elucidating the mechanism related to the pathogenesis of HNSCC, but molecular biomarkers are unavailable for prognosis prediction in the clinic. Thus, we focused on identifying an IHC classifier capable of predicting the prognosis in HNSCC patients. We conducted IHC staining for six immunohistochemical markers (CK, Ki-67, p16, p40, p53, and p63) in paraffin-embedded tumor sections of HNSCC patients. Four markers (CK, Ki-67, p16, and p40) were selected to derive the formula of the IHC score. In prognostic analysis, we found a higher three-year DFS rate in the low IHC score group than in the high IHC score group, which suggested that an increase in the IHC score was associated with poor prognosis. The IHC score, along with age, gender, drinking status, smoking status, TNM stage, and tumor location, were independent prognostic factors in the multivariate Cox model.

Based on previous studies, these IHC markers (CK, Ki-67, p16, and p40) have been implicated in tumor development and progression. Cytokeratins (CKs) are epithelial-specific intermediate filament proteins associated with cell adhesion and migration (23, 24). There is evidence that CKs play an oncogenic role in the proliferation and metastasis of cancer cells. As reported by Jang TH, et al, CK17 activates integrin β4/α6 and increases FAK, Src, and ERK phosphorylation in oral cancer cells to enhance cancer stemness (25). Human melanoma and breast cancer cells with high expression of CK8 and CK18 exhibit higher migration and invasion ability (7, 26). Similarly, our research showed that patients with CK expression had worse survival outcomes than those with low CK expression. Known as the most reliable proliferation marker, Ki-67 is a nuclear non-histone protein that is detected in actively proliferating normal and neoplastic cells (27). Ki-67 immunohistochemistry is considered an important prognostic indicator in oesophageal, lung, kidney, and breast carcinomas (28), which is consistent with our results. The cyclin-dependent kinase inhibitor p16 decreases pRb phosphorylation and inhibits cell cycle progression from G1 to S (29). Deficiency of p16 is a potential druggable target. Overexpression of p16 induced by recombinant adenovirus has significant antitumor effects on human HNSCC (30). In our study, p16 was found to be associated with a better prognosis in HNSCC patients, which is consistent with the findings of these previous studies. ΔNp63 detected by p40 antibody serves as a specific marker for the differentiation of squamous epithelial cells (31). The anti-p40 antibody has gained diagnostic utility in recent years for distinguishing head and neck sarcomatoid squamous cell carcinoma from mesenchymal tumors (32). Several studies demonstrates that ΔNp63 plays a pro-tumorigenic role. For instance, ΔNp63 enhances Wnt signaling by increasing the expression of the Wnt receptor Fzd7, and thereby promotes normal mammary stem cell activity (33). Another major function of ΔNp63 is to inhibit the activity of some tumor suppressor genes, such as p53, TAp63 and TAp73 (34, 35). In line with previous studies, our results showed that HNSCC patients with high p40 expression exhibited poor outcomes. In addition to playing significant roles in tumor initiation and progression, these genes have also been associated with patient outcomes, which makes them suitable for developing a prediction model for prognostic stratification of HNSCC patients. Previous studies have mainly used RNA sequencing or gene expression microarray data to build models. Unlike previous studies, we used immunohistochemical data to construct a prognostic model based on the expression levels of these genes. As one of the most commonly used and convenient methods, immunohistochemical analyses would greatly contribute to the utility and generalizability of the model.

After evaluation of these IHC markers, our results suggested that the IHC score based on the combined expression of the four markers (CK, Ki-67, p16, and p40) can predict survival in HNSCC patients. Kaplan-Meier survival curves supported the strong ability of the IHC score to distinguish cases with high recurrence risk from those with low recurrence risk. Moreover, the IHC score was determined by the univariate analysis along with the multivariate Cox regression analysis to be an independent prognostic factor. Additionally, the ROC analysis showed that the IHC score had stronger predictive power than the TNM stage. Finally, a nomogram was constructed, based on the IHC score, along with additional critical clinicopathological characteristics, for accurately predicting survival in HNSCC patients. After evaluating the calibration curve and the C-index, the results showed that the performance of the nomogram was good.

Our model had several advantages. First, the large study population reinforced the reliability of the results. Second, the IHC scores and other clinical features incorporated into the nomogram were inexpensive and readily available markers. Finally, the nomogram can be used to calculate individualized prognostic estimates without complex mathematical calculations, further demonstrating the clinical utility of the model.

However, our study had some limitations. First, the data were collected from a single institution. Larger studies are needed to confirm our results. Another limitation was that we conducted a retrospective analysis, which might have introduced some bias in the study. Further prospective studies are required to verify the hypothesis.

## Conclusions

We demonstrated that the IHC score based on four immunohistochemical markers, including CK, Ki-67, p16, and p40, was significantly associated with prognosis in HNSCC patients. A nomogram integrating the IHC scores and multiple clinical factors can be a powerful tool for evaluating clinical outcomes in patients with HNSCC.

## Data availability statement

The raw data supporting the conclusions of this article will be made available by the authors, without undue reservation.

## Ethics statement

This study was approved by The Institutional Review Board of Sun Yat-Sen University Cancer Center.

## Author contributions

Study conception and design: Q-QX, Q-JL, and XW. Data acquisition: Q-QX, Q-JL, ZX, L-LL, ZH and JL. Data analysis and interpretation: Q-QX, Q-JL, and ZX. Quality control of data and algorithms: LXL, Y-YC, R-ZC, and XW. Manuscript writing: Q-QX, Q-JL, and XW. Manuscript reviewing and approving: LXL, Y-YC, R-ZC, and XW. All authors have read and agreed to the published version of the manuscript. All authors contributed to the article and approved the submitted version.
